# Gastrointestinal stromal tumour of the duodenum in childhood: a rare case report

**DOI:** 10.1186/1471-2407-7-79

**Published:** 2007-05-09

**Authors:** Massimo Chiarugi, Christian Galatioto, Piero Lippolis, Giuseppe Zocco, Massimo Seccia

**Affiliations:** 1 Department of Surgery, University of Pisa, Pisa, Italy

## Abstract

**Background:**

Gastrointestinal stromal tumours (GISTs) are uncommon primary mesenchymal tumours of the gastrointestinal tract mostly observed in the adults. Duodenal GISTs are relatively rare in adults and it should be regarded as exceptional in childhood. In young patients duodenal GISTs may be a source of potentially lethal haemorrhage and this adds diagnostic and therapeutic dilemmas to the concern about the long-term outcome.

**Case presentation:**

A 14-year-old boy was referred to our hospital with severe anaemia due to recurrent episodes of upper gastrointestinal haemorrhage. Endoscopy, small bowel series, scintigraphy and video capsule endoscopy previously done elsewhere were negative. Shortly after the admission, the patient underwent emergency surgery for severe recurrence of the bleeding. At surgery, a 4 cm solid mass arising from the wall of the fourth portion of the duodenum was identified. The invasion and the erosion of the duodenal mucosa was confirmed by intra-operative pushed duodenoscopy. The mass was resected by a full-thickness duodenal wall excision with adequate grossly free margins. Immunohistochemical analysis of the specimen revealed to be positive for CD117 (c-KIT protein) consistent with a diagnosis of GIST. The number of mitoses was < 5/50 HPF. Mutational analysis for c-KIT/PDGFRA tyrosine kinase receptor genes resulted in a wildtype pattern. The patient had an uneventful course and he has remained disease-free during two years of follow-up.

**Conclusion:**

Duodenal GISTs in children are very rare and may present with massive bleeding. Cure can be achieved by complete surgical resection, but even in the low-aggressive tumours the long-term outcome may be unpredictable.

## Background

Gastrointestinal stromal tumours (GISTs) are uncommon mesenchymal tumours of the gastro-intestinal tract (GI) believed to originate from the interstitial cells of Cajal (ICC) [1]. Likewise ICC, the GIST cells carry the receptor tyrosine kinase (KIT). Immunohistochemically the specific marker for KIT (CD117) is used to distinguish GIST from others spindle-cell tumours of the GI. Although a GIST may arise anywhere in the GI, stomach and small bowel are the most elective sites [2]. It can be estimated that the frequency location of GISTs in the duodenum account approximately to 5% [3-5]. GISTs are far more common in adults, and over the age of 40. A report focused on the epidemiology of GISTs and including 1,458 cases found no patient below the age of 20 [6]. Others large sample size retrospective studies confirm that GISTs are mostly observed in adults and are rare in children. Only 2.7% of GISTs of the stomach and 0.6% of GISTs of the small-bowel occur before the age of 21 years and the mean age of patients presenting with duodenal GISTs is over 50 [3-5]. Duodenal GISTs in children are extremely rare. We report a new clinical observation to enforce knowledge about GISTs of the duodenum in young patients.

## Case presentation

**Figure 1:**
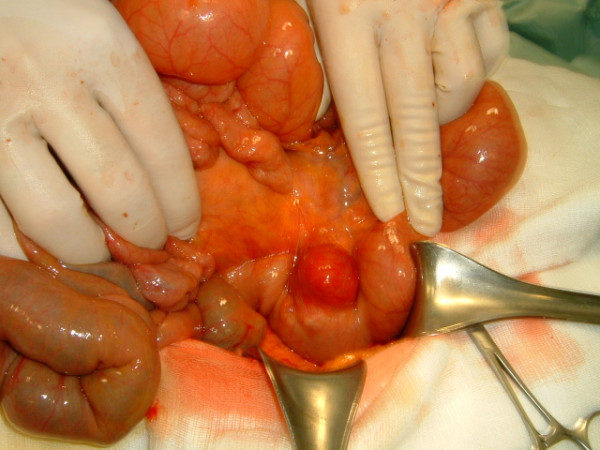
Operative view: esophytic GIST of the fourth duodenal portion.

A 14 year-old boy was referred to the emergency department for severe episodes of upper gastrointestinal bleeding, the first of whom dated back to one year. A few days before, the patient had been admitted elsewhere for melena, tachycardia, mild hypo-tension and severe anaemia (Hb7.5 g/dl). At that time, after blood volume replacement, the patient underwent upper and lower endoscopy studies, abdominal ultrasound (US) scan, pertecnate 99 m (Tc99m) scintigraphy and video-capsule enteroscopy, all of which resulted negative. The patient re-bled severely shortly after his arrival at the emergency department and surgery was warranted. At laparotomy there was fresh blood into the jejunum and ileum but not into the stomach. Exposure of the duodenum by extended Kocher's manoeuvre and Treitz's ligament take-down, allowed the detection of a roundish solid mass, 4 cm. in diameter, arising from the pancreatic border of the fourth portion of the duodenum. The mass, mostly esophytic, did not invade the pancreatic body (Figure 1).

**Figure 2:**
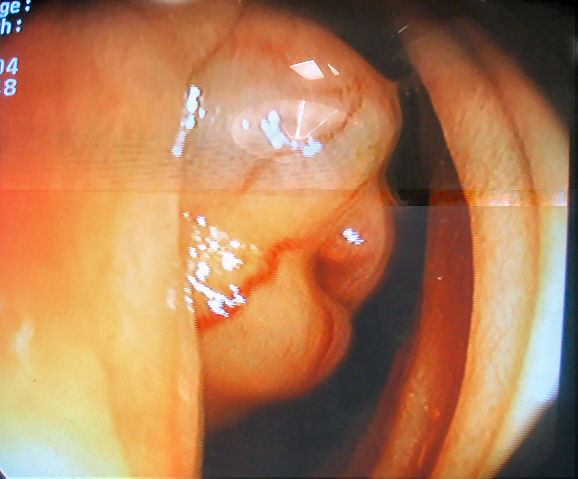
Intra-operative push-endoscopy: GIST protusion into the duodenal lumen with mucosa ulcerated.

Intra-operative pushed upper endoscopy was performed. At endoscopy, the tumour protruded into the lumen and centrally ulcerated the mucosa. (Figure 2) A mild ongoing haemorrhage from the erosion was seen.

The duodenal wall was carefully dissected free from the lower pancreatic border and from the superior mesenteric artery. Removal of the mass was then accomplished by a full-thickness excision of the anterior duodenal wall with adequate grossly free margins. The duodenal defect was over-sewn and covered by an omental patch.

**Figure 3:**
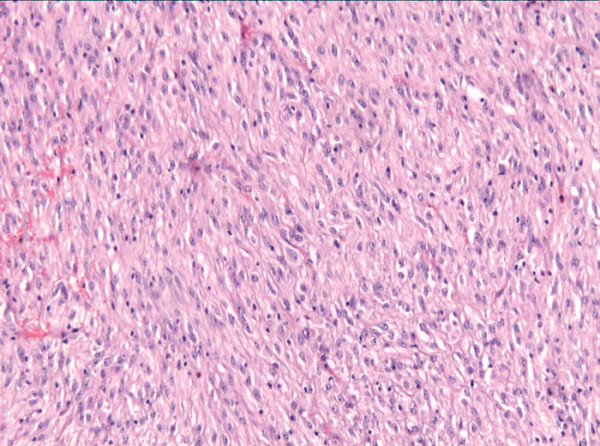
Microscopic appearance of the duodenal GIST demonstrating epithelioid and spindle cells (H&E; magnification, ×10).

**Figure 4:**
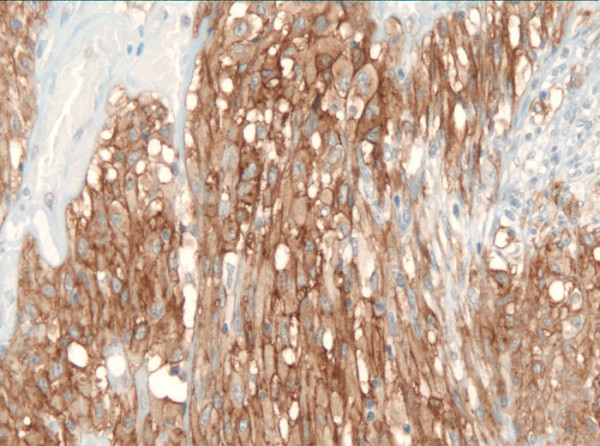
GIST cells stained positive for CD117(c-KIT) (magnification, × 20).

Histopathologic report revealed the tumour was composed by epithelioid and spindle cells (Figure 3), with a mitotic activity < 5 per 50 high power field (HPF). The tumour stained positive for CD117 (Figure 4), CD34 and smooth muscle actine and negative for S100 protein, CK-pan, CD45-LCA, and melanosome, consistent with the diagnosis of GIST. Genomic DNA was isolated from paraffin-embedded tumor tissue. Molecular work-up based on polymerase chain amplification followed by direct sequence analysis of selected coding sequences of KIT (exons 9,11, and 13) and platelet-derived growth factor receptor α (PDGFRα; exons 12,14, and 18) yielded no evidence of mutation. As diagnosis of GIST was made, the patient was investigated to detect clinical features of type 1 neurofibromatosis and of Carney's triad (GIST associated with paraganglioma and pulmonary chondroma). Both syndromes were excluded. In addition, no other family members resulted affected by GIST. The course was uneventful and the patient has remained asymptomatic with no local or distant recurrence at the two years follow-up. No program of imatinib mesylate chemotherapy was undertaken.

## Conclusion

Most GISTs arise in adults over 40 years of age [6]. In the pediatric age group, GISTs occur in the sporadic form and in some cases are associated with Carney's triad or type 1 neurofibromatosis. Rare cases of familial GISts which carry a KIT or PDGFRa germline mutation have been also described [7,8]. The duodenal localization of a GIST in children is however exceptional. Cypriano and Coll. [9] reported in 2004 a series of 7 pediatric patients affected by GIST: none of them originated from the duodenum. More recently, from a review of the English-language biomedical literature, Hayashi & Coll.[10] found 25 children affected by GIST, none of them with a duodenal localization. Two further series of pediatric GISTs, each collecting five cases, failed to identify a duodenal localization [11,12]. So far, the only previously described pediatric patient with a duodenal GIST is by Towu and Coll. that reported in two papers the case of a 7-year-old boy: bleeding was the clinical presentation and the tumour was successfully resected [13,14]. Likewise in adults, duodenal GISTs in children may have an acute clinical onset, with bleeding as one of the most common complication. Endo-luminal bleeding is the expression of local invasion and erosion of the GI mucosa. For GISTs of the foregut, the identification of the source of bleeding may be easily performed by upper endoscopy whenever the tumor is located in the stomach or in the upper duodenum. On the other hand GISTs of the distal duodenum may remain undetected at endoscopy. Alternative diagnostic means for occult upper GI bleeding include duodenum series, US and computed tomography, video-capsule endoscopy, Tc99m scintigraphy and angiography. In the presented cases most of them resulted un-effective in making the diagnosis that was cleared only at surgery. Surgical removal of a duodenal GIST may be accomplished by several options, ranging from the minimal to outmost demolitive procedures. Local tumour excision [15], segmental duodenal resection [16-18] and pancreatoduodenectomy [19-21] have all been advocated, depending on the site and the size of the tumour and on the surrounding organs invasion. Total tumour resection is the standard of cure for GISTs. Nodes dissection is not recommended, because GISTs rarely metastasize to lymph nodes [22]. In eligible patients, total resection of a duodenal GIST can be achieved by a simple excision procedure, that is effective in controlling the bleeding and in achieving cure. Children may benefit of advantages from lesser demolitive duodenal surgery. Treatment with imatinib, an inhibitor of receptor tyrosine kinases (RTKs), may be offered to pediatric patients with advanced GIST disease, not radically amenable by surgery. Several papers have reported a correlation between the nature of molecular alterations of KIT and PDGFRα and response to imatinib treatment [23-25]. In adults most GISTs are caused by mutations in the KIT or PDGFRα receptor tyrosine kinase genes and this make them amenable to therapy with imatinib. Thus, adult patients with unresectable or recurrent GIST have a better prognosis because the disease usually responds to therapy. In children genes mutations have been described in the rare cases of familial GISTs, but they do occur very rarely in sporadic GISTs [11,12,26]. Because most pediatric GISTs have KIT/PDGFRα wildtype patterns, these patients are expected to respond to imatinib treatment less well than adults do.

Complete surgical resection of GISTs seems however more likely in pediatric rather than in adult patients. In a paper presenting a series of pediatric patients with GIST and collecting data from literature, the rate of complete surgical resection was 80% (18 out 23 patients) and more than 90% of these patients survived with no evidence of recurrence [9]. In adults, complete surgical resection of GISTs has been reported in 40% of cases [27]. Moreover the recurrence of GISTs in adults ranges from 40 to 80% despite complete surgical resection of the primary tumor [22,27]. These data suggest a more benign course for pediatric GISTs respect to adult cases.

According to the risk assessment criteria [2], the GIST of the presented case fell in to the low-risk of aggressive behaviour category (tumour size < 5 cm and mitotic counts < 5 mitotic figures per 50 HPF) and no chemotherapy after surgery was offered. Miettinen & Coll. [4] in a review of 156 duodenal GIST including adult and pediatric patients, found that 86% of those with a tumour > 5 cm with > 5 mitoses per 50 HPF died of disease, whereas no recurrence or metastases were seen in patients with tumour < 2 cm with < 5 mitoses/50 HPF. However, they occasionally observed the development of metastases even if the mitotic activity was < 5/50 HPF and the tumour size was < 5 cm. Thus, in young patients, even for the low-aggressive category of totally resected duodenal GISTs, the long-term outcome remains unpredictable. A long-term follow-up study is strongly advisable.

## Competing interests

The author(s) declare that they have no competing interests.

## Authors' contributions

MC conceived of the study and participated in its design and coordination

CG conceived of the study and participated in its design

PL participated in the design of the study and drafted the manuscript

GZ participated in the design of the study and helped in drafting the manuscript

MS conceived of the study and participated in its coordination.

All the Authors read and approved the final manuscript.

## Pre-publication history

The pre-publication history for this paper can be accessed here:
